# Curriculum Mapping as a Tool for Faculty Support, Reflection, and Curricular Integration

**DOI:** 10.1002/jdd.70187

**Published:** 2026-03-02

**Authors:** Enzo Moreira da Silva, Marcela Charantola Rodrigues

**Affiliations:** ^1^ Department of Biomaterials and Oral Biology, School of Dentistry University of São Paulo (FOUSP) São Paulo Brazil

## 1 Problem

Undergraduate dental curricula are in a continuous process of reform, aiming to integrate scientific content with clinical practice and strengthen competency‐based learning [1]. While these changes promote coherence and relevance, they also create uncertainty among faculty members about their teaching scope, learning objectives, and curricular responsibilities. Such uncertainty may lead to frustration and weaken the teaching–learning process. In 2023, the School of Dentistry, University of University of São Paulo (FOUSP) implemented a new integrated curriculum, revealing these challenges within the Dental Biomaterials discipline. Faculty members reported difficulty identifying where and how their content would appear in the revised curriculum. To address this and to promote pedagogical reflection during the transition to an integrated program, a structured curriculum mapping project was designed as both a support and diagnostic tool. This initiative sought to help faculty realign their content and strengthen curricular coherence during implementation of the new curriculum.

## 2 Solution

In response to these challenges, we conducted a curriculum mapping of Dental Biomaterials content based on Harden's [2] framework. Six faculty members (five full‐time and one part‐time instructors) participated, all from the Department of Biomaterials and Oral Biology, representing both preclinical and laboratory teaching activities. The process included document analysis (syllabi, pedagogical project, and class schedules) and semi‐structured interviews exploring faculty perceptions of content, learning outcomes, and teaching methodologies [3]. Interview transcripts were analyzed through an inductive thematic process aligned with Harden's curriculum mapping dimensions (learning outcomes, content, teaching methods, and assessment).

**FIGURE 1:**
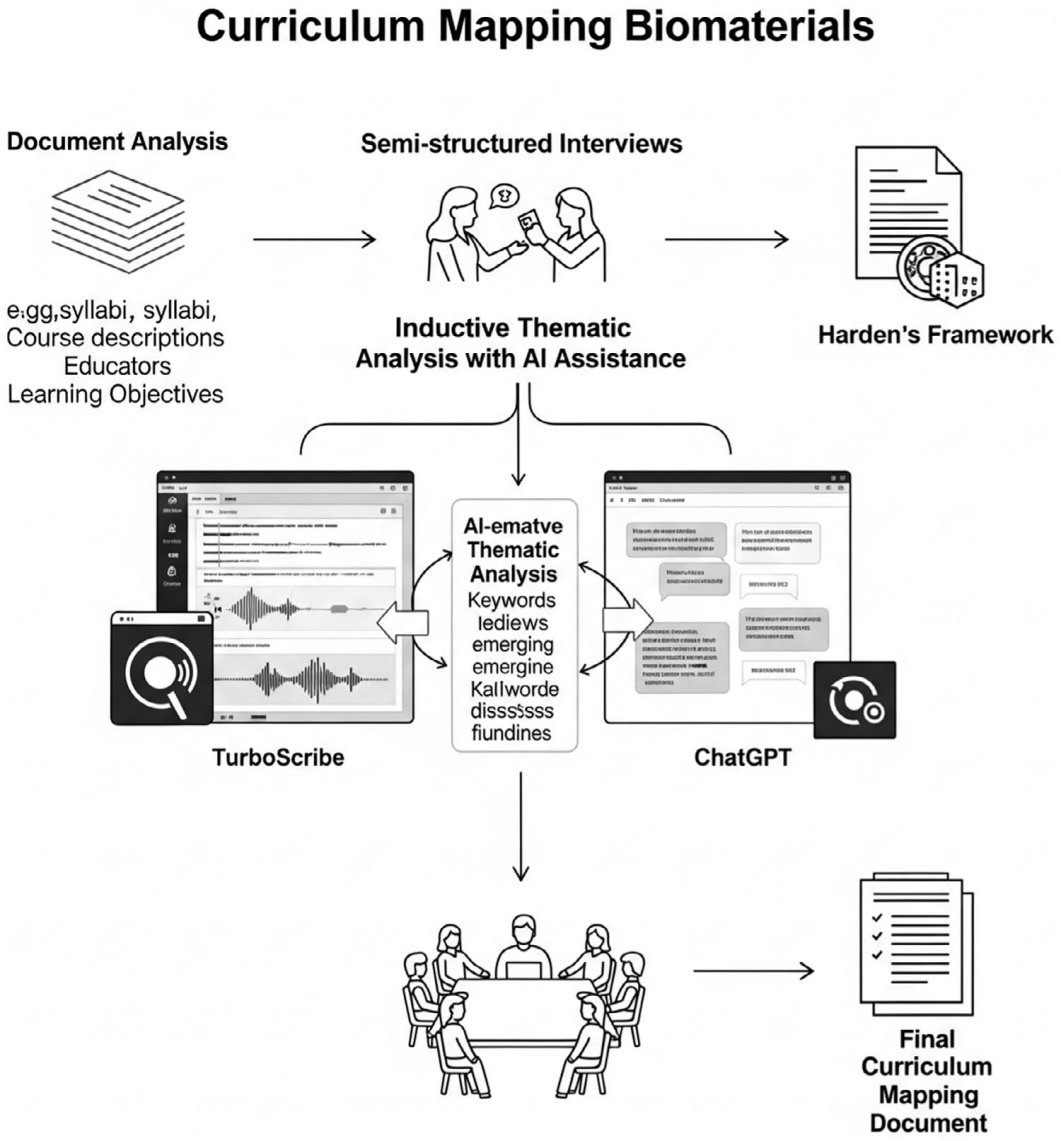
Workflow of the Dental Biomaterials Curriculum Mapping. This structured process, based on Harden's framework, involved document analysis and semi‐structured interviews with faculty. Data analysis used Inductive Thematic Analysis, streamlined by AI tools. All outputs underwent human validation by the interviewed professors, resulting in the Final Curriculum Mapping document.

Artificial intelligence tools were incorporated to enhance efficiency: TurboScribe was used for transcription and ChatGPT supported synthesis and language refinement. All outputs were subsequently reviewed and validated by the interviewed faculty members to ensure accuracy, reliability, and ethical integrity. The final mapping document detailed the location, objectives, and pedagogical approaches of each content area and was shared with the department chair as a reference for curricular coordination and faculty development Figure 1.

## 3 Results

The curriculum mapping revealed a redistribution of Dental Biomaterials content, with increased laboratory workload, improved articulation between theoretical and practical sessions, and reduced overlap with other disciplines. Faculty reflections indicated that the process fostered greater pedagogical awareness and collaborative dialogue, allowing them to reconsider their educational intentions and align teaching strategies with program‐level competencies. These effects align with previously described benefits of curriculum mapping, particularly regarding improved transparency and coherence across health professions curricula [4]. Concrete outcomes included the integration of laboratory exercises earlier in the curriculum, the identification of redundant theoretical topics, and the recognition of opportunities for interdepartmental collaboration—findings consistent with prior evaluations of curriculum mapping within dental materials education [5]. Challenges also emerged, such as limited faculty time to assimilate changes, continued reliance on lecture‐based teaching, and initial resistance to modifying long‐established content. Despite these constraints, the process functioned as both a managerial and reflective instrument, enhancing curricular clarity and supporting faculty in navigating the transition to an integrated program.1
